# A Melanin-Related Phenolic Polymer with Potent Photoprotective and Antioxidant Activities for Dermo-Cosmetic Applications

**DOI:** 10.3390/antiox9040270

**Published:** 2020-03-25

**Authors:** Davide Liberti, Maria Laura Alfieri, Daria Maria Monti, Lucia Panzella, Alessandra Napolitano

**Affiliations:** Department of Chemical Sciences, University of Naples “Federico II”, Via Cintia 4, I-80126 Naples, Italy; davide.liberti@unina.it (D.L.); marialaura.alfieri@unina.it (M.L.A.); alesnapo@unina.it (A.N.)

**Keywords:** melanins, 5,6-dihydroxyindole-2-carboxylic acid, antioxidant, photoprotection, UVA, HaCaT cells, reactive oxygen species, glutathione, Nrf-2

## Abstract

Eumelanins, the dark variant of skin pigments, are endowed with a remarkable antioxidant activity and well-recognized photoprotective properties that have been ascribed to pigment components derived from the biosynthetic precursor 5,6-dihydroxyindole-2-carboxylic acid (DHICA). Herein, we report the protective effect of a polymer obtained starting from the methyl ester of DHICA (MeDHICA-melanin) against Ultraviolet A (UVA)-induced oxidative stress in immortalized human keratinocytes (HaCaT). MeDHICA-melanin was prepared by aerial oxidation of MeDHICA. At concentrations as low as 10 µg/mL, MeDHICA-melanin prevented reactive oxygen species accumulation and partially reduced glutathione oxidation in UVA-irradiated keratinocytes. Western blot experiments revealed that the polymer is able to induce the translocation of nuclear factor erythroid 2–related factor 2 (Nrf-2) to the nucleus with the activation of the transcription of antioxidant enzymes, such as heme-oxygenase 1. Spectrophotometric and HPLC analysis of cell lysate allowed to conclude that a significant fraction (ca. 7%), consisting mainly of the 4,4′-dimer of MeDHICA (ca. 2 μM), was internalized in the cells. Overall these data point to the potential use of MeDHICA-melanin as an antioxidant for the treatment of skin damage, photoaging and skin cancers.

## 1. Introduction

**Figure 1:**
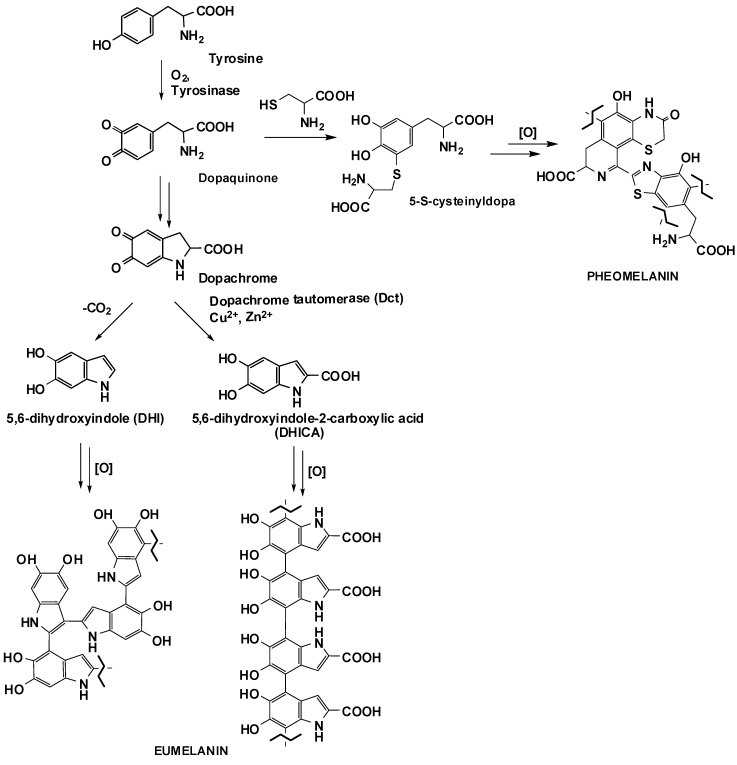
Biosynthetic pathways leading to eumelanins and pheomelanins.

Melanins are the primary determinants of skin, hair and exoskeletal pigmentation in mammals, birds and insects [1,2,3,4,5,6], and their importance has been growing over the past few years not only from a biological point of view, related to their role in the human body, but also for the exploitation of their unique properties in biomedicine or in the cosmetic and health sectors [7,8,9,10,11,12,13,14,15,16]. These pigments are biosynthesized in melanocytes, starting with the oxidation of tyrosine to dopaquinone catalyzed by the enzyme tyrosinase (Figure 1) [1,17,18]. Dopaquinone may then undergo cyclization leading, after a further oxidation step, to 5,6-dihydroxyindole (DHI) or 5,6-dihydroxyindole-2-carboxylic acid (DHICA) whose oxidative polymerization ultimately leads to melanin pigments brown or dark in color, known as eumelanins [1,9,18]. On the other hand, entrapment of dopaquinone by cysteine, a process which is under genetic control, gives rise to isomeric cysteinyldopas whose polymerization is responsible for the biosynthesis of the reddish-brown pigments known as pheomelanins, typical of the red hair phenotype [1,19,20] (Figure 1). 

Traditionally, eumelanins have been attributed a role as antioxidant and photoprotective agents in dark-skinned phenotypes, whereas pheomelanins have been implicated in the enhanced susceptibility to skin cancer of individuals belonging to the red-hair phenotype due to their photosensitizing and pro-oxidant properties [1,9,21,22,23,24,25,26]. Among eumelanins, bionspired synthetic pigments obtained by oxidative polymerization of DHICA have shown remarkable antioxidant properties, and have been proposed as a plausible explanation for the high content of DHICA-related units in natural eumelanins [27,28]. Indeed, DHICA-melanin is able to act as a potent hydroxyl radical scavenger in the Fenton reaction [29] and has been found to act as an efficient antioxidant and radical scavenger also in the 2,2-diphenyl-1-picrylhydrazyl (DPPH), 2,2′-azinobis(3-ethylbenzothiazoline-6-sulfonic acid) (ABTS) and nitric oxide scavenging assays [30]. DHICA-melanin also exhibited inhibition properties against in vitro lipid peroxidation [31], whereas silica/DHICA-melanin hybrid nanoparticles exerted protecting effects against hydrogen peroxide-induced cytotoxicity [32]. More recently, the higher antioxidant activity of DHICA-melanin compared to DHI-melanin has also been confirmed by the Folin–Ciocalteu assay [33]. 

The antioxidant properties of DHICA-melanin seem to also play a role in the maintenance of immune hyporesponsiveness to melanosomal proteins of relevance for the onset of autoimmune vitiligo [34]. Recently, a natural pigment isolated from marine *Aspergillus nidulans* and tentatively identified as a DHICA-melanin exhibited protective effects against Ultraviolet B (UVB)-induced oxidative stress in cellular and mice models [35,36]. UV radiations are known to be very harmful for the human skin, as they induce reactive oxygen species (ROS) production. Both Ultraviolet A (UVA) and UVB are able to induce DNA damages [37,38]. UVB radiations induce DNA dimerization reactions between adjacent pyrimidine bases, whereas UVA radiations, weakly absorbed by DNA, can excite endogenous chromophores, leading to mispairing of DNA bases with consequent translation of mutated proteins [39].

In this context, the remarkable antioxidant properties of DHICA-melanin and its chromophoric characteristics, allowing significant absorption in the UVA region [1,9,30], would suggest its use in dermo-cosmetic formulations with photoprotective action.

**Figure 2:**
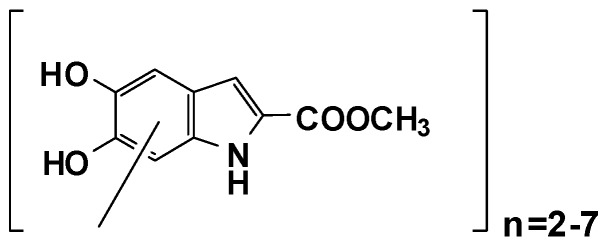
Structure proposed for the methyl ester of 5,6-dihydroxyindole-2-carboxylic acid (MeDHICA)-melanin based on MALDI-MS analysis [42].

Yet, full exploitation of DHICA-melanin has so far been hampered by the low solubility in lipophilic or hydroalcoholic solvents usually employed in cosmetics, and the relatively high susceptibility to (photo)degradation [40,41]. On these bases, we recently developed a variant of DHICA-melanin which was obtained by oxidative polymerization of the methyl ester of DHICA (MeDHICA-melanin) and shown to consist of a collection of intact oligomers from the dimer up to the heptamer by MALDI-MS analysis (Figure 2) [42]. The material was characterized by an intense and broad absorption band centered at 330 nm and proved to be soluble in water miscible organic solvents. Moreover, MeDHICA-melanin retained the antioxidant properties of DHICA-melanin, proving indeed even more active. It was also stable to prolonged oxidation or exposure to a solar simulator [42].

Here, we report the protective effect of MeDHICA-melanin, prepared by aerial oxidation of MeDHICA, on oxidative photodamage of immortalized human keratinocytes (HaCaT) induced by UVA-exposure. Keratinocytes represent the most exposed cellular layer in the epidermis, functioning as a protective barrier from environmental stimuli, pathogens and radiation, and it is now generally recognized that molecules endowed with antioxidant activity, especially polyphenols, can strengthen the barrier function of keratinocytes from photoaging [43,44].

## 2. Materials and Methods

### 2.1. Reagents

MeDHICA was prepared as described in [42]. MeDHICA-melanin was prepared by aerial oxidation of MeDHICA in phosphate buffer at pH 8.5, as previously reported [42]. Phosphate buffer saline (PBS), Dulbecco’s modified Eagle’s medium (DMEM), fetal bovine serum (HyClone), 3-(4,5-dimethylthiazol-2-yl)-2,5-diphenyltetrazolium bromide (MTT), 2′,7′-dichlorodihydrofluorescein diacetate (H_2_DCFDA), l-glutamine, trypsin-EDTA, Triton, 5,5′-dithiobis-2-nitrobenzoic acid (DTNB), thiobarbituric acid (TBA), and Bradford reagent were from Sigma-Aldrich (St. Louis, MI, USA). Bicinchoninic acid (BCA) protein assay kit was from Thermo Scientific (Waltham, MA, USA). Antibodies against nuclear factor erythroid 2–related factor 2 (Nrf-2) and heme oxygenase 1 (HO-1) were from Cell Signal Technology (Danvers, MA, USA). Antibodies against B-23 and β-actin and the chemiluminescence detection system (SuperSignal^®^ West Pico) were from Thermo Fisher Scientific (Waltham, MA, USA).

### 2.2. Cell Culture

Human immortalized keratinocytes (HaCaT) were from Innoprot (Derio, Spain). Cells were cultured in DMEM, supplemented with 10% fetal bovine serum, 2 mM l-glutamine and antibiotics in a 5% CO_2_ humidified atmosphere at 37 °C. Every 48 h, cells were refreshed in a ratio 1:5. The culture medium was removed and cells were rinsed with PBS and then detached with trypsin-EDTA. After centrifugation (5 min at 1000 rpm), cells were diluted in fresh medium.

### 2.3. Analysis of Cell Viability

Cells were seeded in 96-well plates (100 µL/well) at a density of 2.5 × 10^3^ cells/cm^2^. 24 h after seeding, cells were incubated with increasing concentrations (0.1, 1, 5 and 10 µg/mL) of MeDHICA-melanin. Mother solutions of MeDHICA-melanin were prepared in DMSO at a concentration of 0.2 mg/mL and proper aliquots were added to the incubation medium to get the desired concentration. After 24 h and 48 h incubation, cell viability was assessed by the MTT assay. The MTT reagent, dissolved in DMEM without phenol red, was added to the cells (0.5 mg/mL). After 4 h at 37 °C, the culture medium containing MTT was removed and the resulting formazan salt was dissolved in 2-propanol containing 0.01 M HCl (100 µL/well). Absorbance values of blue formazan were determined at 570 nm using an automatic plate reader (Microbeta Wallac 1420, Perkin Elmer, Milano, Italy). Cell survival was expressed as the percentage of viable cells in the presence of MeDHICA-melanin compared to the controls, represented by untreated cells and cells supplemented with identical volumes of DMSO, in order to exclude a possible effect of DMSO on cell viability.

### 2.4. UVA irradiation and H_2_DCFDA Assay

To evaluate the protective effect of MeDHICA-melanin against oxidative stress, cells were plated at a density of 3.5 × 10^4^ cells/cm^2^ (in 6o mm eukaryotic cell plates), pre-incubated in the presence of increasing concentration (0.1–10 µg/mL) of MeDHICA-melanin for different lengths of time (from 5 to 120 min) and stressed by UVA light for 10 min (100 J/cm^2^) [45]. Then, cells were incubated with the cell permeable, redox-sensitive fluorophore H_2_DCFDA at a concentration of 20 µM for 30 min at 37 °C. Cells were then washed with cold PBS 2 times, detached by trypsin, centrifuged at 1000 rpm for 10 min and resuspended in PBS containing 30 mM glucose, 1 mM CaCl_2_, and 0.5 mM MgCl_2_ (PBS plus) at a cell density of 1 × 10^5^ cells/mL. H_2_DCFDA is nonfluorescent until it is hydrolyzed by intracellular esterases, and in the presence of ROS it is readily oxidized to the highly fluorescent 2′,7′-dichlorofluorescein (DCF). DCF fluorescence intensity was measured at an emission wavelength of 525 nm with excitation wavelength set at 488 nm using a Perkin-Elmer LS50 spectrofluorometer (Perkin Elmer, Milano, Italy). Emission spectra were acquired at a scanning speed of 300 nm/min, with 5 slit widths for excitation and emission. ROS production was expressed as percentage of DCF fluorescence intensity of the sample under test, with respect to the untreated sample.

### 2.5. Western Blot Analysis

HaCaT cells were plated at a density of 3.5 × 10^4^ cells/cm^2^ (100 mm eukaryotic cell plates) in complete medium for 24 h and then treated with 10 µg/mL of MeDHICA-melanin for 5, 15, 30 and 60 min. To extract nuclear proteins, cells were first incubated with PBS buffer containing 0.1% Triton and protease inhibitors to extract cytosolic proteins. After centrifugation at 1200 rpm for 10 min, nuclear pellet was obtained and proteins were extracted by resuspending the pellet in radioimmunoprecipitation assay buffer (RIPA) buffer (150 mM NaCl, 1% NP-40, 0.1% SDS, proteases inhibitors in 50 mM Tris-HCl pH 8.0). Proteins were quantified by BCA protein assay kit. Western blotting was used to analyze 100 µg of proteins, as reported [46]. Nuclear factor erythroid 2–related factor 2 (Nrf-2) and heme oxygenase 1 (HO-1) levels were detected by using specific antibodies. To normalize protein intensity levels, specific antibodies against B-23 and β-actin were used for nuclear and cytosolic extracts, respectively. Signals were detected by using the chemiluminescence detection system.

### 2.6. Catalase Assay

HaCaT cells were plated at a density of 3.5 × 10^4^ cells/cm^2^ (in a 100 mm eukaryotic cell plate) in complete medium for 24 h and then treated with 10 µg/mL of MeDHICA-melanin for 60 min. At the end of the experiment, total cell lysate was obtained by resuspending each cell pellet in 50 µL of lysis buffer (100 mM Tris-HCl, 300 mM NaCl and 0.5% NP-40 at pH 7.4 with addition of inhibitors of proteases and phosphatases). Proteins were quantified by BCA protein assay kit. To measure catalase activity, a procedure reported in the existing literature was followed [47]. Briefly, cell lysates (50 µg of proteins) were incubated for 30 min at room temperature in 1 mL of hydrogen peroxide solution (50 mM potassium phosphate buffer, pH 7.0, 0.036% *w/w* H_2_O_2_). Then, the hydrogen peroxide concentration in solution was determined by measuring the absorbance at 240 nm. The percentage of peroxide removed was calculated as following:

% H_2_O_2_ reduced = 1 − OD_240nm_ sample/OD_240nm_ standard

Standard is referred to the hydrogen peroxide solution in the absence of lysate and measured at 240 nm after 30 min of incubation.

### 2.7. Determination of Intracellular Glutathione (GSH) Levels

HaCaT cells were plated at a density of 3.5 × 10^4^ cells/cm^2^ (60 mm eukaryotic cell plates) in complete medium for 24 h and then treated with 10 µg/mL of MeDHICA-melanin for 60 min. At the end of the photoirradiation experiment, cells were lysed, and protein concentration was determined by the Bradford colorimetric assay. Proteins (50 µg) were incubated in the presence of 3 mM EDTA, 144 µM DTNB in 30 mM Tris-HCl at pH 8.2, and centrifuged at 13,000 rpm for 5 min at 4 °C. Supernatants were collected, and the absorbance was measured at 412 nm by using a multiplate reader (Bio-Rad, Hercules, CA, USA). GSH levels were expressed as % of the sample under test with respect to the untreated sample.

### 2.8. Analysis of Lipid Peroxidation Levels

HaCaT cells were seeded at a density of 3.5 × 10^4^ cells/cm^2^ (100 mm eukaryotic cell plates) in complete medium for 24 h and then treated with 10 µg/mL of MeDHICA-melanin for 120 min. After UVA irradiation, cells were kept at 37 °C for 90 min, before performing the thiobarbituric acid reactive substances (TBARS) assay as described [48]. Briefly, cells were detached and 5 × 10^4^ cells suspended in 0.67% TBA containing 20% trichloroacetic acid (TCA) (1:1 *v/v*). After heating for 30 min at 100 °C, samples were centrifuged at 2500 rpm for 5 min at 4 °C, and supernatants spectrophotometrically analyzed at 532 nm. 

### 2.9. Quantification of Internalized Melanin

HaCaT cells were plated at a density of 3.5 × 10^4^ cells/cm^2^ (100 mm eukaryotic cell plates) in complete medium for 24 h and then treated with 10 µg/mL of MeDHICA-melanin for 60 min. After treatment, total cell lysate was obtained, 50 µg of proteins (Bradford assay) were diluted in 1 mL of potassium phosphate buffer (50 mM, pH 7.0) and UV-vis spectra were recorded. The amount of MeDHICA-melanin internalized by the cells was determined by using a calibration curve obtained with pure MeDHICA-melanin. In particular, increasing concentrations (0.6–20 µg/mL) of MeDHICA-melanin, alone or in the presence of 50 µg of cell lysate, were used to record the UV-vis spectra. The calibration curve was built by plotting values of absorbance at 330 nm against MeDHICA-melanin concentration. 

### 2.10. HPLC and LC-MS Analysis of Cell Lysate

HPLC analysis was performed on an instrument (Agilent 1100, Santa Clara, CA, USA) equipped with a binary pump and a SPD-10AV VP UV-vis detector set at 300 nm. The chromatographic separation was achieved on a Sphereclone octadecylsilane-coated column, 250 mm × 4.6 mm, 5 μm particle size (Phenomenex, Torrance, CA, USA) at 0.7 mL/min using binary gradient elution conditions as follows: 0.1% formic acid (solvent A), acetonitrile (solvent B) from 35% to 70%, 0–45 min. LC-MS analyses were run on a LC-MS ESI-TOF 1260/6230DA Agilent instrument operating in positive ionization mode in the following conditions: Nebulizer pressure 35 psig; drying gas (nitrogen) 5 L/min, 325 °C; capillary voltage 3500 V; fragmentor voltage 175 V. An Eclipse Plus C18 column, 150 × 4.6 mm, 5 μm (Agilent), at a flow rate of 0.4 mL/min was used, using the same eluant as above. The cell lysate, obtained as described in Section 2.9, was lyophilized and subjected to acetylation treatment with acetic anhydride (500 μL) and pyridine (75 μL) overnight. After repeated washings with methanol to remove solvents, the residue was taken up in methanol and analyzed by HPLC and LC-MS. A control lysate sample obtained in the absence of MeDHICA-melanin was also analyzed. 

### 2.11. Statistical Analysis

In all the experiments, each sample was tested in three independent analyses, each carried out in triplicate. The results are presented as mean of results obtained (mean ± SD) and compared by one-way ANOVA following Tukey’s multiple comparison test using Graphpad Prism for Windows, version 6.01 (San Diego, CA, USA).

## 3. Results and Discussion

### 3.1. Biocompatibility of MeDHICA-Melanin on Keratinocytes

**Figure 3:**
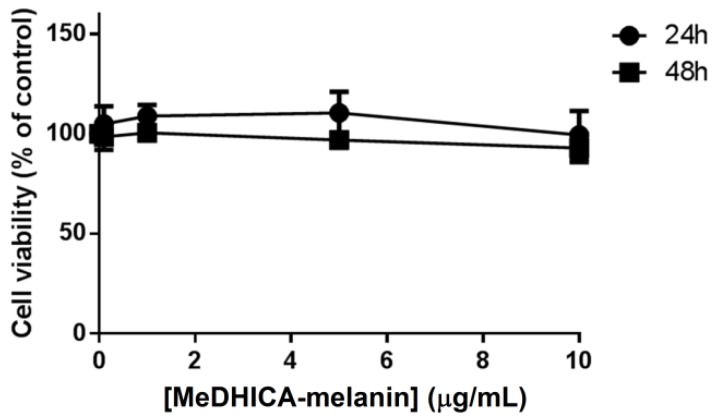
Effects of MeDHICA-melanin on HaCaT cells viability. Dose-response curves after 24 h (black circles) and 48 h (black squares) incubation of HaCaT cells with increasing concentration of MeDHICA-melanin (0.1–10 µg/mL). Cell viability was assessed by the MTT assay and cell survival expressed as percentage of viable cells in the presence of MeDHICA-melanin, with respect to control cells (i.e., cells grown in the absence of the melanin). The results shown are means ± SD of three independent experiments.

In order to assess the possible use of MeDHICA-melanin for cosmetic applications, its biocompatibility was tested on immortalized human keratinocytes (HaCaT), as these cells are normally present in the outermost layer of the skin. Increasing amounts of MeDHICA-melanin (from 0.1 to 10 µg/mL) were incubated with the cells for 24 and 48 h. At the end of each incubation, cell viability was assessed by the MTT assay. As shown in Figure 3, cell viability was not affected at any of the experimental conditions tested, neither after 24 h nor after 48 h incubation, thus suggesting that MeDHICA-melanin was fully biocompatible on HaCaT cells. 

### 3.2. Inhibition of UVA-Induced Damage on HaCaT Cells by MeDHICA-Melanin

To assess the protective effect of MeDHICA-melanin against photoinduced oxidative stress, irradiation with UVA was chosen as a source of stress as this has been shown to induce many side effects on human skin [49]. A dose-response experiment was first performed to evaluate the optimal MeDHICA-melanin concentration to be used. HaCaT cells were incubated with increasing concentrations of MeDHICA-melanin (0.1–10 µg/mL) for 2 h prior to UVA irradiation treatment, and immediately after irradiation ROS production was evaluated by the H_2_DCFDA assay.

**Figure 4:**
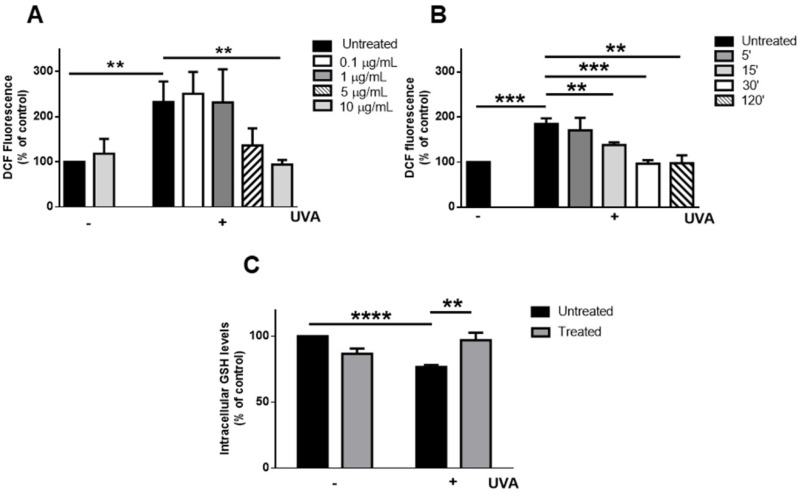
Antioxidant effects of MeDHICA-melanin on UVA-stressed HaCaT cells. (**A**) Dose-response analysis of intracellular ROS levels by 2’,7’-dichlorodihydrofluorescein diacetate (H_2_DCFDA) assay. Cells were pre-incubated with increasing concentrations of MeDHICA-melanin for 2 h prior to UVA irradiation (100 J/cm^2^) for 10 min. Cells were incubated with 0.1 µg/mL (white bars), 1 µg/mL (dark grey bars), 5 µg/mL (dashed bars) or 10 µg/mL (light grey bars) MeDHICA-melanin. Black bars refer to untreated cells. (**B**) Time-course analysis of intracellular ROS levels by H_2_DCFDA assay. Cells were incubated for 5 min (dark grey bars), 15 min (light grey bars), 30 min (white bars) or 120 min (dashed bars) with MeDHICA-melanin before being irradiated by UVA. Black bars are referred to untreated cells. (**C**) Intracellular GSH levels evaluated by 5,5′-dithiobis-2-nitrobenzoic acid (DTNB) assay. Cells were pre-incubated with MeDHICA-melanin (10 µg/mL) for 1 h before UVA irradiation. Values are expressed as % with respect to control (i.e. untreated) cells. Data shown are means ± SD of three independent experiments. ** indicates *p* < 0.005, *** indicates *p* < 0.0005, **** indicates *p* < 0.0001.

As shown in Figure 4A, DCF fluorescence was significantly increased after UVA irradiation (2.3 fold increase, *p* < 0.005), whereas MeDHICA-melanin had no effect on ROS levels on non-irradiated cells. Interestingly, when cells were preincubated with MeDHICA-melanin prior to UVA exposure, ROS production was decreased in a dose dependent manner, and reached the levels observed in non-irradiated cells when the melanin was tested at 10 µg/mL (*p* < 0.005) (Figure 4A). 

The effect of the preincubation time with MeDHICA-melanin on ROS production was also evaluated (Figure 4B). A significant protection against UVA damage was observed already after 15 min of incubation with 10 µg/mL of MeDHICA-melanin (*p* < 0.005). These data confirmed the potent antioxidant activity of MeDHICA-melanin [42] also in a cellular model, further highlighting its potential as an active ingredient in cosmetic formulations when compared to other natural or synthetic materials, such as phenol-rich plant extracts or other melanin-related samples. As an example, 10-fold higher concentrations (100 μg/mL) and longer pre-incubation times (1 h) have been reported in the case of a water extract from red grapevine leaves containing high levels of polyphenols to observe an effect comparable to that of the present study on the decrease of ROS generation in HaCaT cells irradiated with lower doses of UVA (25 J/cm^2^) [38]. Also, the activity of silymarin was much lower than that observed with MeDHICA-melanin: 30 min of pre-incubation with 250 μg/mL of the compound were able to reduce the ROS produced by irradiating HaCaT cells with 20 J/cm^2^ UVA by only 30% [50].

Based on these promising results, subsequent experiments were carried using 10 µg/mL MeDHICA-melanin.

The intracellular levels of GSH were evaluated in view of the important role of this biomolecule in the cellular redox balance, and the decrease associated to oxidative stress [51]. Following UVA irradiation, a 25% decrease (*p* < 0.0001) of intracellular GSH levels was observed with respect to control cells, whereas GSH levels were unaltered in cells preincubated with MeDHICA-melanin (Figure 4C). Similar effects have been reported on UVA-irradiated HaCaT cells for phenol-rich extracts from *Eugenia uniflora* [52] and *Syzygium aqueum* [53] leaves, which, however, had to be tested at higher concentrations (50 μg/mL) and for a longer time (2 h) to show a protective effect. 

The different behavior of all these samples compared to MeDHICA-melanin can be ascribed to differences in the chemical structures of the compounds tested, determining crucial variations not only in the intrinsic antioxidant activity, but also in the cell-permeation ability as well as in the UVA-interaction properties.

The protective effects of MeDHICA-melanin on HaCaT cells were further confirmed by analyzing the lipid peroxidation levels 90 min after irradiation (Figure S1). The results indicated that MeDHICA-melanin was able to keep lipid peroxidation unaltered. In fact, cells pretreated with MeDHICA-melanin and then exposed to UVA radiation showed a significantly lower intracellular level of lipid peroxidation when compared to untreated cells exposed to UVA (50% decrease, *p* < 0.005) (Figure S1). Notably, a lower protective effect against lipid peroxidation has been reported for the well-recognized antioxidant pterostilbene in 20 J/cm^2^ UVA irradiated-HaCaT cells after 24 h pretreatment with 2.5 μg/mL of the compound [54]. However, a significant increase in lipid peroxidation levels was observed in cells incubated only with MeDHICA-melanin (2.4 fold increase), without photoirradiation, an intriguing observation that will be addressed in future studies. In any case, the overall results clearly indicate that MeDHICA-melanin is able to protect HaCaT cells from UVA-induced oxidative stress.

### 3.3. Induction of Nrf-2 Nuclear Translocation by MeDHICA-melanin

**Figure 5:**
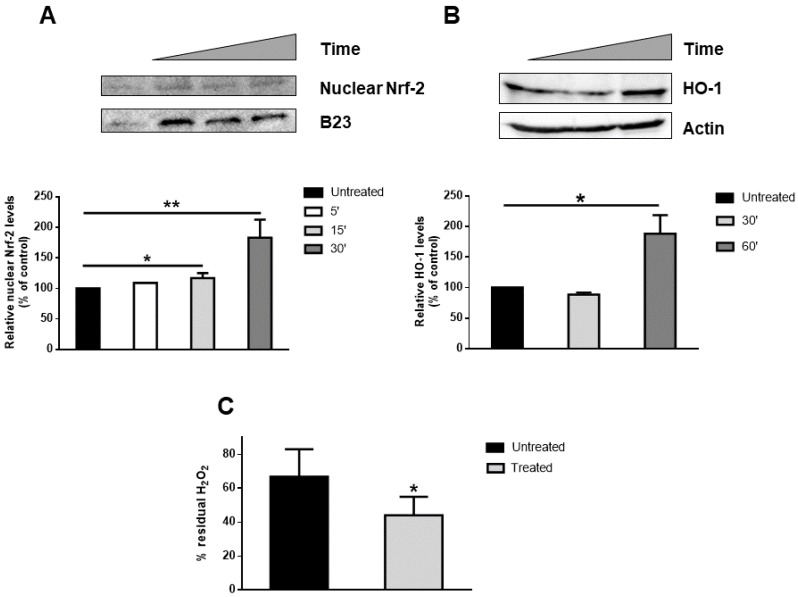
MeDHICA-melanin effects on Nrf-2 activation in HaCaT cells. Cells were incubated with MeDHICA-melanin (10 µg/mL) for different lengths of time, and (**A**) nuclear Nrf-2, or (**B**) cytosolic HO-1 proteins were analyzed by Western blotting. (**A**) HaCaT cells were incubated with MeDHICA-melanin for 5 min (white bars), 15 min (light grey bars) and 30 min (dark grey bars) and then nuclear proteins extracted to perform Western blot analysis of Nrf-2. Nrf-2 was quantified by densitometric analysis and normalized to B-23. (**B**) Western blot analysis for HO-1 performed on cytosolic proteins obtained from HaCaT cells after incubation with MeDHICA-melanin for 30 min (light grey bars) and 60 min (dark grey bars). HO-1 was quantified by densitometric analysis and normalized to β-Actin. (**C**) Cells were incubated with melanin (10 µg/mL) for 1 h and then 50 µg of cell lysate were incubated with 0.036% w/w H_2_O_2_. Hydrogen peroxide concentration in solution was determined by measuring the absorbance at 240 nm. Black bars are referred to control cells. Data shown are means ± SD of three independent experiments. * indicates *p* < 0.05 ** indicates *p* < 0.005.

The MeDHICA-melanin protective effect was analyzed at a molecular level by studying the involvement of Nrf-2. Under normal physiological conditions, the complex between Nrf-2 and Keap-1 keeps Nrf-2 in the cytosol and the protein is degraded through the proteasome. Oxidative stress, or small amounts of antioxidants, induce the dissociation between Keap-1 and Nrf-2, and the latter is translocated to the nucleus. Once in the nucleus, it binds to antioxidant responsive element (ARE) sequences and activates the transcription of several phase-II detoxifying enzymes, such as HO-1 and catalase [55]. Thus, cells were incubated with MeDHICA-melanin for 5, 15 and 30 min and then nuclear Nrf-2 levels were evaluated by Western blot analyses. As shown in Figure 5A, a significant increase of Nrf-2 nuclear levels was observed after 30 min of incubation (about 2 fold increase, *p* < 0.005). Nrf-2 activation was confirmed by measuring HO-1 levels (Figure 5B), which were found to significant increase after 60 min incubation of the cells with MeDHICA-melanin (about 2 fold increase, *p* < 0.05). Nrf-2 activation was also confirmed by measuring consumption of H_2_O_2_ added to the incubation medium, that could indirectly indicate the activity of catalase. As reported in Figure 5C, the levels of H_2_O_2_ detected in keratinocyte lysates were lower (33% decrease, *p* < 0.05) in the cells after incubation with MeDHICA-melanin with respect to the control sample. 

### 3.4. Cellular Uptake of MeDHICA-Melanin

**Figure 6:**
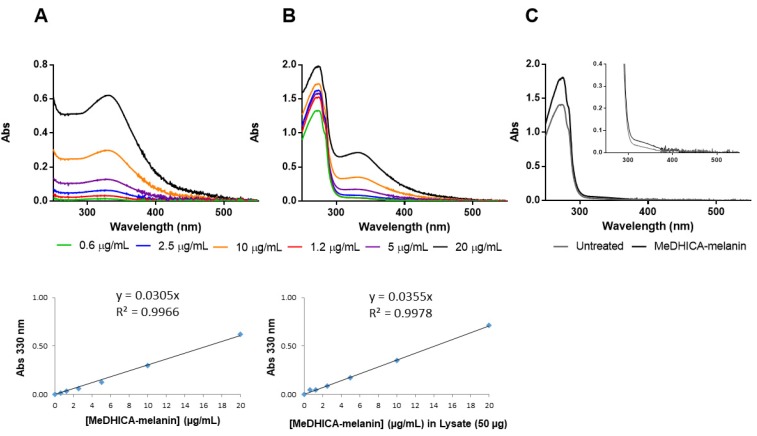
Quantification of internalized MeDHICA-melanin. Increasing concentrations (0.6–20 µg/mL) of MeDHICA-melanin, (**A**) alone, or (**B**) in the presence of 50 µg of cell lysate, were used to record the UV–vis spectra. Calibration curves built by plotting values of absorbance at 330 nm against MeDHICA-melanin concentration are also shown. (**C**) UV–vis spectra of untreated cells (grey line) and MeDHICA-melanin treated (black line) HaCaT cells.

In order to verify whether MeDHICA-melanin was internalized in the cells, HaCaT cells were incubated with MeDHICA-melanin at 10 μg/mL for 60 min, after that the total cell lysate was obtained. UV–vis spectra of lysates from untreated and treated cells were recorded and the amount of melanin internalized by the cells was estimated to be about 7% using a measurement of the absorbance at 330 nm of treated cells lysate and a comparison with the calibration curve obtained by using pure MeDHICA-melanin (Figure 6). This result is in agreement with the by now well-established idea that melanin is internalized by cells to serve as a protective agent [56].

**Figure 7:**
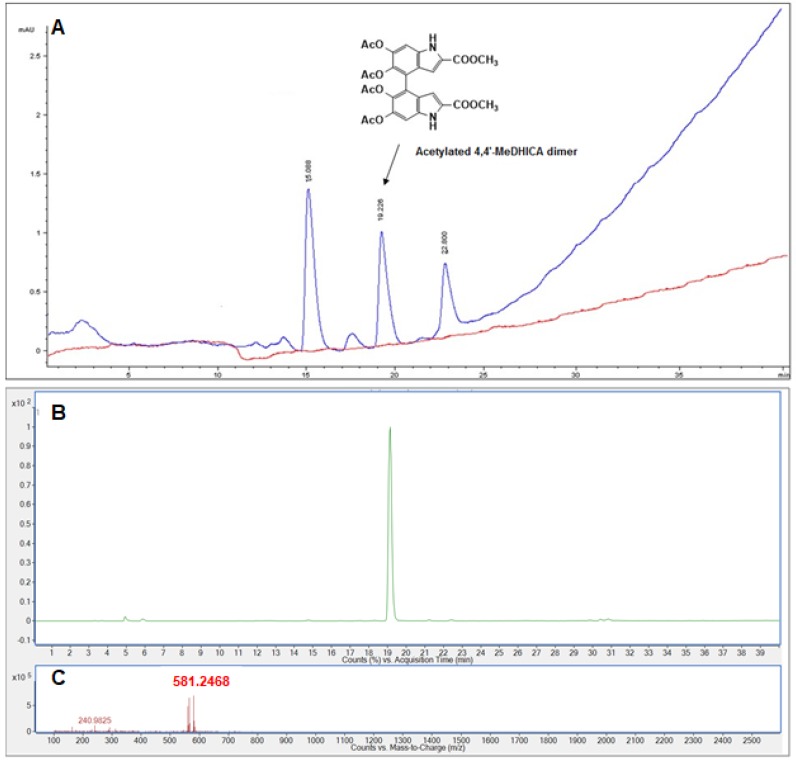
HPLC and LC-MS analysis of cell lysate. (**A**) HPLC profile of acetylated cell lysate (blue trace) and acetylated lysate from control cells (not incubated with MeDHICA-melanin) (red trace). (**B**) LC-MS extracted ion chromatogram (*m/z* 581). (**C**) MS spectrum of the compound eluted at 19 min.

MeDHICA-melanin internalization was further corroborated by HPLC analysis of the cell lysate upon incubation with the pigment. To improve the chromatographic properties and the stability, the cell lysate obtained by HaCaT incubation with 10 μg/mL of MeDHICA-melanin was acetylated with acetic anhydride-pyridine overnight at room temperature and then analyzed by HPLC (Figure 7A).

Comparison of the elutographic properties with those of an authentic standard [42] allowed to identify the compound eluted at 19 min as the acetylated derivatives of the 4,4′-dimer of MeDHICA (ca. 2 μM). The identity of this product was further confirmed by LC-MS analysis ([M+H]^+^ for acetylated dimer = 581 *m/z*) (Figure 7B,C).

## 4. Conclusions

UVA radiations are highly harmful as they can penetrate the skin, crossing the epidermis and reaching the dermis. They are responsible for a variety of physiopathological conditions, ranging from inflammation to premature skin aging and skin cancer development [45,57,58]. On the other hand, the antioxidant and photoprotective properties of eumelanins and model pigments from DHICA are well-established [29,30,41,42].

In this paper, MeDHICA-melanin, previously shown to possess marked in vitro antioxidant activity and favorable solubility properties for dermo-cosmetic applications [42], was demonstrated to exert protective effects on a cellular model of immortalized keratinocytes (HaCaT) exposed to UVA radiations. All the endpoint parameters of oxidative stress, i.e., ROS, lipid peroxidation, and intracellular oxidized glutathione levels, were significantly inhibited in cells incubated with MeDHICA-melanin at concentrations compatible with cell viability. Moreover, a comparison with related findings recently reported in the literature on natural phenols or phenol-rich extracts further highlighted the advantages of MeDHICA-melanin. 

Similarly to other natural antioxidants [58], MeDHICA-melanin also proved to provide protection by activating the Nrf-2 pathway. Indeed, the nuclear translocation of Nrf-2 was effective, as demonstrated by the activation of downstream genes. To our knowledge, this is the first report on the correlation between the antioxidant activity of melanins and the Nrf-2 pathway. We also demonstrated that MeDHICA-melanin was able to enter keratinocytes, although, as expected, only 

Low molecular weight components such as dimeric compounds were appreciably internalized after 1 h incubation. These data provide further confirmation to biological studies indicating that keratinocytes are able to internalize melanins as the result of a cross-talk with melanocytes, mediated by the protease-activated receptor 2 (PAR-2) and the Ras-related protein Rab11 [59,60]. Overall, our findings demonstrate that MeDHICA-melanin is able to enter the cells and activate the antioxidant system to protect skin cells from UVA-induced damage, encouraging its use as an effective component in dermo-cosmetic formulations for the treatment of skin damage, photoaging and skin cancers.

## Supplementary Materials

The following are available online at https://www.mdpi.com/2076-3921/9/4/270/s1, Figure S1: Lipid peroxidation levels evaluated by TBARS assay.
