# Supplementary material for: A Melanin-Related Phenolic Polymer with Potent Photoprotective and Antioxidant Activities for Dermo-Cosmetic Applications
Source: Antioxidants (Basel). 2020 Mar 25;9(4):270. doi: 10.3390/antiox9040270 (PMC7222206; doi:10.3390/antiox9040270)
Supplement: Supplementary file 1 [file antioxidants-09-00270-s001.pdf]

Supplementary Materials

A Melanin-Related Phenolic Polymer with Potent Photoprotective and Antioxidant Activities for Dermo-Cosmetic Applications

Davide Liberti, Maria Laura Alfieri, Daria Maria Monti,* Lucia Panzella * and Alessandra Napolitano

Department of Chemical Sciences, University of Naples "Federico II", Via Cintia 4, I-80126 Naples, Italy; davide.liberti@unina.it (D.L.); marialaura.alfieri@unina.it (M.L.A.); alesnapo@unina.it (A.N.)

* Correspondence: mdmonti@unina.it (D.M.M.); panzella@unina.it (L.P.); Tel.: +39-081-679150 (D.M.M.); +39-081-674131 (L.P.)

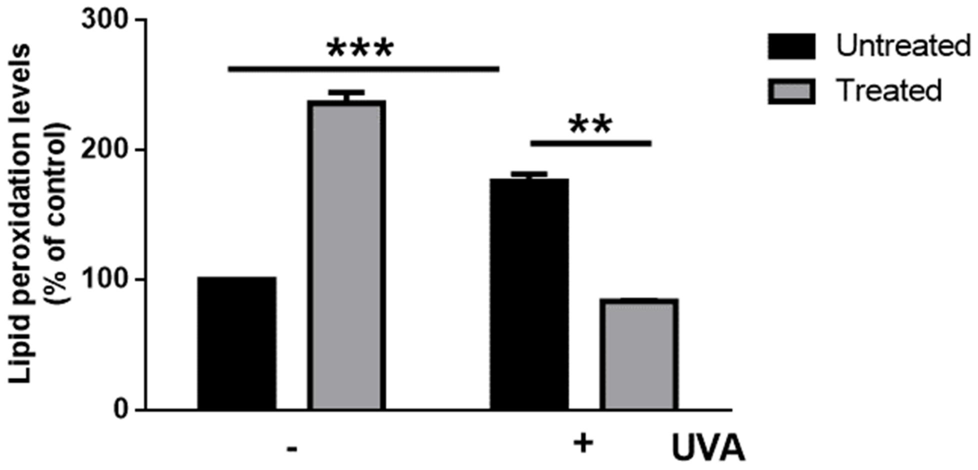

Figure S1. Lipid peroxidation levels evaluated by TBARS assay. Cells were pre-incubated with MeDHICA-melanin (10 μg/mL) for 2 h before UVA irradiation and then incubated for 90 min. Values are expressed as % with respect to control (*i.e.* untreated) cells. Data shown are means ± SD of three independent experiment. ** indicates $p < 0.005$, *** indicates $p < 0.0005$. Black bars refer to control cells and grey bars to cells incubated with MeDHICA-melanin. (−) indicates non irradiated cells; (+) indicates cells exposed to UVA irradiation.
